# Ceiling of Care in Acute Care Settings: A Concept Analysis

**DOI:** 10.1155/jonm/6624553

**Published:** 2026-09-22

**Authors:** Hussein Al-Qarni, Hadwan Aldahmashi, Della Maneze, Luke Molloy, Peta Drury, Yenna Salamonson

**Affiliations:** ^1^ School of Nursing, University of Wollongong, Wollongong, New South Wales, Australia, uow.edu.au; ^2^ Department of Adult Nursing, College of Nursing, King Khalid University, Abha, Saudi Arabia, kku.edu.sa; ^3^ College of Nursing, University of Hafr Albatin, Hafr Albatin, Saudi Arabia; ^4^ School of Nursing and Midwifery, Western Sydney University, Penrith, Australia, westernsydney.edu.au; ^5^ Australian Centre for Integration of Oral Health (ACIOH), Liverpool, Australia

## Abstract

**Aim(s):**

To conduct a systematic concept analysis of Ceiling of Care to clarify its definition and distinguish its core features within acute care settings.

**Method:**

The Walker and Avant’s concept analysis method was applied.

**Data Sources:**

A systematic search of six databases (APA PsycINFO, CINAHL, the Cochrane Library, MEDLINE, ProQuest Central and Scopus) was conducted from inception to July 2026, supplemented by manual reference list searches and AI‐assistance tools.

**Result:**

Nineteen records met the inclusion criteria. Five defining attributes were identified: (1) patient‐centred collaborative decision‐making; (2) comprehensive and structured prognostic evaluation; (3) establishment of explicit therapeutic boundaries; (4) proactive and continuous care strategy and (5) standardised documentation and communication. Antecedents included predisposing, precipitating and contextual factors, while key consequences included reduced nonbeneficial treatment and enhanced decision‐making clarity. The analysis distinguishes Ceiling of Care as a broad, anticipatory framework rather than a reactive, single‐intervention directive such as a do‐not‐resuscitate order.

**Conclusions:**

Ceiling of Care is defined as an anticipatory, documented clinical decision‐making approach that establishes the maximum appropriate level of treatment escalation for an individual patient, based on clinical assessment, anticipated benefit and patient values.

**Implication for Nursing Management:**

This conceptual clarity provides a foundation for standardised implementation in acute care settings. Nurse managers need to lead the integration of Ceiling of Care frameworks within acute care settings; this could support nursing management in standardising clinical decision‐making, reducing nonbeneficial treatment escalation, strengthening communication across multidisciplinary teams and ultimately enhancing the quality and dignity of patient care.

## 1. Introduction

Determining the appropriate limits of treatment escalation remains one of the most significant challenges in acute care practice [1]. Clinicians need to navigate a complex landscape where prognostic uncertainty and the potential therapeutic benefit of ongoing treatment are weighed against patients’ or families’ expressed values and preferences, often within the practical constraints of the healthcare system, institutional policies and available resources [2, 3]. The stakes of these decisions are high, as aggressive escalation may subject patients to burdensome interventions that cause harm without meaningful benefit [4], while overly cautious withholding of therapy may deny patients a viable opportunity for recovery [5]. Despite rapid advances in life‐sustaining technologies, many healthcare settings still lack a unified policy for defining these treatment boundaries [6]. While various frameworks exist, such as the treatment‐escalation plan (TEP) and the do‐not‐resuscitate (DNR), significant confusion persists regarding their meanings and applications. This terminological inconsistency, combined with the conceptual nebulousness surrounding how and when these terms are applied in practice, often undermines the clinical clarity these frameworks were intended to provide.

In this context, the concept of Ceiling of Care has emerged to describe a predefined upper limit of appropriate therapeutic intervention for an individual patient [7]. Ideally, this process is proactive and multidimensional, encompassing open discussions between patients, families and the multidisciplinary team before a crisis occurs. It represents a deliberate shift away from traditional medical paternalism toward a collaborative, values‐based model of care planning [8]. When patients are supported in defining their own Ceiling of Care while they retain decision‐making capacity, the resulting plan is more likely to be aligned with their personal goals, values and tolerance for risk [9]. However, despite its reference in the literature for nearly two decades [10], the concept remains poorly defined and is routinely conflated with related but distinctive directives such as advance care plan, TEP or DNR [7]. Ceiling of care and other treatment‐limitation approaches share an interest in aligning treatment with clinical circumstances and patient preferences, but differ in scope, timing and clinical function. A DNR order applies specifically to cardiopulmonary resuscitation [11], while advance care planning is a broader process for identifying values, goals and future treatment preferences [12]. Treatment‐escalation approaches document clinically appropriate interventions and limitations for a current episode of care [13]. In contrast, Ceiling of Care provides an anticipatory and clinically actionable statement of the maximum appropriate level of treatment escalation while also clarifying which treatments remain appropriate [7]. These distinctions are summarised in Table 1. This conceptual ambiguity limits its practical utility and weakens its potential to transform treatment escalation into a process of genuine shared decision‐making.

**TABLE 1:** Comparison of ceiling of care with traditional orders.

Feature	Ceiling of care	Advance care planning (ACP)	Do not resuscitate (DNR)	Treatment‐limiting decisions
Timing	Anticipatory (before crisis)	Anticipatory (before crisis)	Prior to potential arrest	Often reactive (during crisis)
Scope	Broad framework for all care	Specific to future treatment	Specific to cardiac arrest	Specific to single therapy
Primary goal	Values‐aligned guidance	Values‐aligned guidance	Withholding cardiopulmonary resuscitation	Stopping nonbeneficial care
Documentation	Standardise and integrated	The broad aim of treatment	Specific order	Clinical notes

The complexity of these decisions is particularly acute outside of specialised environments like intensive care. In critical care settings, established frameworks, such as those guiding the withdrawal of life‐sustaining therapies, provide structured processes that reduce moral stress and ensure transparency [14]. In contrast, the general acute care environment often lacks such clear policy guidance [15]. Here, sudden clinical deterioration or conflicts between family wishes and medical recommendations can heighten anxiety of those involved and exacerbate clinician moral distress [16].

The objective of this study is to address the persistent conceptual ambiguity surrounding the term Ceiling of Care by clarifying its definition and distinguishing features in acute care contexts. Using Walker and Avant [17] method of concept analysis, we systematically synthesise the literature to identify the defining attributes, antecedents and consequences of the concept. By establishing a clear operational definition, this analysis provides a foundation for improving clinical documentation, enhancing interdisciplinary communication and informing robust policy development. Given that nurses are central to care coordination within acute care settings, clarifying this concept and its implications for nursing practice is essential for supporting consistent, ethically sound, patient‐centred decision‐making.

## 2. Methods

This study combined a systematic literature search with a formal concept analysis to ensure methodological rigour and conceptual depth. The systematic search was reported in accordance with the Preferred Reporting Items for Systematic Reviews and Meta‐Analyses (PRISMA) 2020 guidelines [18], aimed to identify all relevant uses and interpretations of the term ‘Ceiling of Care’ across the literature. Because the purpose of this study was to clarify and refine the underlying concept rather than merely summarising existing evidence, a systematic review alone was insufficient. Walker and Avant’s [17] eight‐step method of concept analysis, a widely recognised approach in nursing and health sciences, was therefore employed to interpret the findings and derive a clear, contextually relevant operational definition. This method supports the identification of defining attributes, antecedents and consequences and facilitates the differentiation of Ceiling of Care from related but distinct concepts such as end‐of‐life care, DNR and Do Not Intubate (DNI) orders [11]. Walker and Avant [17] eight‐step method guided the analysis and development of the Ceiling of Care concept. Although the steps are presented sequentially in Table 2, the process was iterative and adaptable. The review followed a predefined protocol registered with the Open Science Framework (OSF; DOI: 10.17605/OSF.IO/PEHA8).

**TABLE 2:** Steps of Walker and Avant’s concept analysis of ceiling of care.

Step	Content
1. Select a concept	Ceiling of Care
2. Determine the aims or purpose of analysis	To clarify its definition and distinguishing features
3. Identify all uses of the concept that can be discovered	Definition and uses of Ceiling of Care
4. Determine the defining attributes	Characteristics that were most frequently associated with ‘Ceiling of Care’
5. Identify a model case	Example of Ceiling of Care that includes all the attributes
6. Identify borderline, related, contrary, invented and illegitimate cases	Other examples of Ceiling of Care that encompass some of or not identified attributes
7. Identify antecedents and consequences	Events that occur prior to Ceiling of Care and the outcomes of Ceiling of Care
8. Define empirical referents	Empirical measures of Ceiling of Care

*Note:* For this review, Ceiling of Care is understood as a predetermined maximum level of clinical intervention considered appropriate for a patient during their acute care episode.

### 2.1. Search Strategy

A comprehensive search was conducted in November 2025 across six databases: APA PsycINFO, CINAHL, the Cochrane Library, MEDLINE, ProQuest Central and Scopus. Individualised search strategies were developed for each database, utilising its specific indexing terms such as Medical Subject Headings (MeSH), along with Boolean operators and truncations. Databases were searched from inception to November 2025, and the results were limited to peer‐reviewed articles published in English. Following the initial search, a supplementary search was conducted using the same databases, extending the search period to July 2026 and using a Boolean structure and adding Nurs∗ to the second concept group. To maximise retrieval, search terms were developed and refined with an expert librarian (RL), focussing on three main concept groups: Ceiling of Care that includes terms capturing its construct and operationalisation (‘Ceiling of Care’ OR ‘ceiling of treatment’ OR ‘withholding treatment’ OR ‘treatment escalation’ OR ‘treatment escalation plan∗’ OR ‘treatment limit∗’ OR ‘advance∗ care plan∗’ OR ‘advance∗ treatment plan∗’); decision‐making, reflecting the decision process, ethical framing, relational models of decision‐making and nursing involvement within this process (‘decision making’ OR discussion∗ OR collaborat∗ OR decision OR ethic∗ OR intervention∗ OR ‘patient centred’ OR Nurs∗); and acute care settings (inpatient∗ OR ‘acute setting∗’ OR ward). In addition, manual forward and backward citation searches were undertaken to identify further relevant studies. Two artificial intelligence tools, Litmaps and ResearchRabbit, were used to double‐check forward citations and minimise the risk of missing key articles. No date restrictions were applied to allow a comprehensive review of relevant literature across all time periods.

### 2.2. Inclusion and Exclusion Criteria

Inclusion criteria comprised articles that addressed Ceiling of Care, advanced care, or limitation or escalation of treatment within adult hospital settings other than critical care. To account for terminological variability across healthcare systems, conceptually related terms (e.g. TEPs, treatment‐limitation decisions and advance care planning) were included to help clarify the conceptual boundaries of Ceiling of Care. Studies focused exclusively on paediatric populations, community or hospice services, palliative or end‐of‐life care, or intensive care unit (ICU) only contexts were excluded. ICU‐specific studies were excluded because these settings have well‐established escalation frameworks [14], which differ substantially from the more challenging decision‐making processes in general acute care [19]. Additionally, conference abstracts, discussion papers and non‐English publications were excluded.

### 2.3. Search Screening

Three authors (HAQ, HAD and YS) conducted the initial search. After importing the results into EndNote 21 and removing duplicates, two authors (HAQ and HAD) independently screened the titles and abstracts. Full texts of potentially relevant studies were retrieved and evaluated against the inclusion criteria by the same independent reviewers. Reasons for exclusion were systematically documented in accordance with PRISMA guidance. At full‐text review, studies were excluded when they focused on end‐of‐life or palliative care, did not address the concept, included paediatric populations or were available only as conference posters. Disagreements mainly revolved around whether studies on related treatment‐limitation practices provided sufficient data on the Ceiling of Care and study settings to satisfy the inclusion criteria. These disputes were resolved through discussions with a third author (YS) until a consensus was achieved.

**FIGURE 1:**
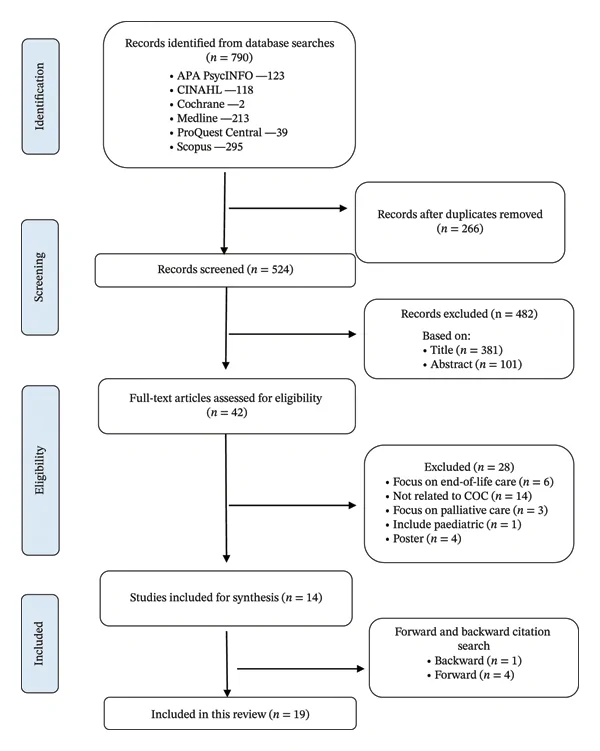
PRISMA flowchart for the study selection and inclusion process.

From an initial 790 articles, 14 met the inclusion criteria. Supplementary searching yielded five additional articles, including one via backward citation searching, three via forward citation searching and one through AI‐assisted searching, resulting in a total of 19 included articles. The complete study‐selection process and reasons for exclusion are presented in the PRISMA flow diagram (Figure 1).

### 2.4. Data Extraction

Data were extracted using a customised form that recorded author, year, country, setting, aim, study design, population, terminology and findings related to the attributes, antecedents and consequences. The characteristics and key findings of the included studies are summarised in Table 3. Data extraction was conducted by the first author (HAQ) and reviewed by coauthors (HAD and YS) to ensure completeness and consistency, and to support a robust characterisation of the concept. Although a formal risk‐of‐bias assessment was not undertaken, consistent with the aims of concept analysis, the research team considered study design, context and methodological clarity when interpreting and weighing each study’s contribution to the conceptualisation of the Ceiling of Care. Additionally, the strength of evidence supporting each attribute was qualitatively appraised according to consistency across sources, direct relevance to Ceiling of Care, breadth across study designs and clinical contexts, and the presence of contradictory or contextually limited findings.

**TABLE 3:** Summaries of included studies.

Author/country/setting	Aim	Method	Finding		
			Attribute	Antecedents	Consequences
Author Byambasuren et al. [52] Country Australia Setting Tertiary hospital (ED and wards)	To assess adherence to local policy recommending advance care planning (ACP) in all hospitalised adult patients with suspected or proven COVID‐19, and to explore the nature and extent of ACP, as well as factors associated with poorer rates of ACP.	Study design Retrospective cohort study Population Hospitalised adults with suspected/proven COVID‐19 (*n* = 209) Terms used in the article Advance care planning	• Patient values elicitation • Formal documentation • Dynamic, ongoing and reversible planning • Time‐sensitive and early decision‐making • Shared, multiparty decision‐making	• Advanced age • Frailty status • High comorbidity burden • Acute deterioration • Pandemic context • Resource constraints	• Improved patient/family satisfaction with the treatment plan • Treatment preference concordance • Cost reduction • Reduced aggressive treatment • Earlier palliative care access • Improved quality of life • Better family experience through communication with care providers • Enhanced family preparedness for expectations surrounding death

Author Coisy et al. [26] Country France and Belgium Setting ED	To find the 30‐day mortality rate of patients who had a treatment withholding or withdrawal decision in the emergency department, focussing on those discharged alive. A secondary aim was to see whether any factors predicted survival in patients with a medium level of treatment withholding or withdrawal.	Study design Retrospective, single‐centre (monocentric) observational study Population Adult ED patients with a decision. 280 patients were included, and 219 (78%) were analysed. Terms used in the article Treatment withholding or withdrawal	• A shared decision needing two senior doctors from different wards • Requires documentation in the medical file • Graded by level (low, medium, high) • Dynamic and revisable (TWW level could be changed in the ward) • Based on the patient’s and family’s wishes • Linked with older age, frailty and comorbidity	• Advanced age (median 85 years) • Frailty status • High comorbidity burden • Acute life‐threatening illness on ED arrival • Pandemic context (COVID‐19 during the study period) • ED overcrowding and workload	• 30‐day survival of 36% (not immediate death) • Avoidance of unreasonable or aggressive care • Placement in a suitable care setting (ward, ICU, palliative care unit or home) • Earlier access to palliative care • The ED decision shaping the rest of the hospital stay • Need for a well‐organised care pathway • Need for regular review of the patient’s condition

Author Cruz‐Carreras et al. [47] Country United States Setting ED of a cancer care centre	To explore the challenges faced in the advanced care planning of cancer patients in an emergency setting and elucidate the difficulties encountered with patients in discussions regarding care through three case presentations.	Study design Case report Retrospective chart review and clinical case descriptions Population Advanced cancer patients (*n* = 3) Terms used in the article Advance care planning	• Requires patient capacity • Formal documentation • Centrality of patient wishes • Surrogate decision‐maker designation • Ethically and legally binding on physicians • Dynamic, ongoing and reversible planning	• Advanced cancer diagnosis • Treatment‐refractory disease • Progressive disease • Poor prognosis • Multiple comorbidities • Acute deterioration	• Family disagreement regarding final decisions or acceptance of the patient’s wishes • Failure of the surrogate to make decisions on behalf of the patient • Promotes shared decision‐making

Author De Decker et al. [53] Country France and Belgium Setting EDs of general hospitals	To determine whether the Charlson Comorbidity Index (CCI) was associated with the treatment‐limiting decisions made for older patients who die in the EDs.	Study design Prospective cross‐sectional population‐based study Population Older adults (≥ 65 years) (*n* = 2095) Term used in the article Treatment‐limiting decision	• Predetermined choice • Made for therapies that would otherwise be deemed necessary • Clinically appropriate care ceiling	• Advanced age • High comorbidity burden • Multiple comorbidities • Functional impairment • Functional limitations	• Increase the rate of death in the ED • Withholding life‐sustaining therapies • Higher mortality in older adults

Author Haimovich et al. [54] Country United States Setting EDs of General Hospitals	To evaluate the ACP engagement of patients ≥ 65 years in the ED using a previously validated instrument and to assess participants’ preferences for learning about ACP topics and their favoured learning formats.	Study design Prospective cross‐sectional study Population Older adults (≥ 65 years) (*n* = 99) Term used in the article Advance care planning	• Communication‐driven decision‐making • Formal documentation	• Advanced age • Frailty • Multiple comorbidities • Functional limitations	• Increased peacefulness at the end of life • Wishes known and followed • Improved ACP engagement

Author Holmes et al. [55] Country United Kingdom Setting District general hospitals and one University hospital EDs	To see whether the treatment escalation or limitation plan (TELP) was used appropriately for patients at the hospital and whether the presence of a TELP had influenced, or would have influenced, out‐of‐hours clinical decision‐ making in patients who were deteriorating, as determined by an advanced nurse practitioner in the HECT.	Study design Prospective evaluation study Population Hospitalised adult inpatients (*n* = 50) Term used in the article treatment escalation or limitation plan (TELP)	• Communication‐driven decision‐making • Clinically appropriate care ceiling • Actionable clinical order • Anticipatory planning • Deterioration‐responsive planning • Extends beyond DNACPR • Formal documentation • Part of ACP	• Acute deterioration • Multiple comorbidities • High risk of death	• Improved communication • Reduced medical harm • Guided clinical decision‐making • Appropriate treatment provision • Prevented inappropriate escalation • Reduced nonbeneficial interventions • Reduced moral distress • Prevents futile treatment

Author Jackson et al. [21] Country United States Setting The Medical Oncology Solid Tumour Service	To test whether an education programme that helped nurses and social workers start ACP earlier would improve care for patients with advanced cancer.	Study design Quality‐improvement project using a pre‐ and postintervention design Population Adults (over 18 years) with a solid tumour diagnosis *n* = 159 Staff surveyed were the unit’s registered nurses and social workers. Term used in the article advance care planning	• A process, not a single event (best done over several patient and provider meetings) • Produces documentation (an advance directive) • Dynamic and revisable across admissions • Time‐sensitive and best started early • Multiparty (patients, families, nurses, social workers, oncology providers) • Supports care that matches patient values and goals	• Advanced or incurable cancer • Risk of sudden decline or loss of capacity • Clinical trial participation with short admissions • Provider reluctance or lack of time and training • ACP and AD being optional	• Care that matches patient preferences • Smoother, shorter care transitions (a trend toward shorter length of stay for patients with an AD) • Better quality end‐of‐life care • Greater patient and family satisfaction • Better chance of dying in the preferred setting • Reduced risk of hasty or unwanted care during a crisis

Author Knight et al. [56] Country United Kingdom Setting Acute medical emergencies of acute hospitals	To establish the prevalence of ACP on initial presentation to the hospital with a medical emergency within SAMBA and to see whether factors such as age or readmission within 30 days affected the availability of ACP.	Study design National cross‐sectional audit/survey Population Frail, older patients with multiple long‐term health conditions (*n* = 6072) Term used in the article Advance care planning	• Involves discussion with family and healthcare providers • Formal documentation • Patient values elicitation • Personalised care planning • Complex process over time	• Progressive disease • Need for ongoing treatment • High disease burden • Treatment risks	• Increased discussion of preferences around care plan • Increased concordance between documented preferences and care received • Reduced family stress • Reduced emergency visits • Lower in‐hospital deaths

Author Lim et al. [57] Country Multiple countries Setting District general hospitals and one university hospital EDs	To determine whether advance care planning in haemodialysis patients, compared with no or less structured forms of advance care planning, can result in fewer hospital admissions or less use of treatments with life‐prolonging or curative intent, and if patient’s wishes were followed at end‐of‐life	Study design Systematic review Population People with end stage of kidney disease undergoing haemodialysis (*n* = 337) Term used in the article Advance care planning	• Anticipatory planning • Requires patient capacity • Multiparty involvement • Communication‐driven decision‐making • Formal documentation • Dynamic, ongoing and reversible Planning • Includes surrogate designation • Personalised care planning	• Progressive disease • Need for ongoing treatment • High disease burden • Treatment risks	• Improved outcomes • Preferences honoured • Increased satisfaction • Reduced family stress • Enhanced patient control • Better concordance between patients’ wishes and treatment plan

Author Misselbrook et al. [31] Country United Kingdom Setting Medical and orthopaedic inpatient wards	To implement and improve engagement with the Recommended Summary Plan for Emergency Care and Treatment (ReSPECT) process and improve consistency and quality of documentation within medical and orthopaedic departments	Study design Quality‐improvement project with pre‐ and postintervention audits Population Frail, older adult inpatients (*n* = 112) Term used in the article ReSPECT	• Personalised care planning • Anticipatory planning • Complementary to the wider ACP • Replaces/extends beyond DNACPR • Formal documentation • Provides specific clinical guidance • Shared, multiparty decision‐making • Dynamic, ongoing and reversible planning • Personalised care planning	• Frailty • High mortality risk • Poor functional outcomes • Multiple comorbidities • High comorbidity burden: ‘median Charlson Comorbidity Index was 7’ • Acute deterioration	• Early anticipatory care planning • Aids immediate decision‐making • Improved form completeness • Improved clinical guidance • Reduced the anxiety of doctors about discussing patients’ situations with colleagues

Author Pallares et al. [24] Country Spain Setting Inpatient wards of general hospitals	To compare, by Ceiling of Care, clinical features and outcomes of hospitalised subjects during four waves of COVID‐19 in a metropolitan area in Catalonia	Study design Observational multicentre cohort study Population Hospitalised adults with proven COVID‐19 (*n* = 5813) Term used in the article Therapeutic Ceiling of Care	• Resource‐contingent decision‐making • Physician‐determined	• Pandemic context • Resource constraints • Advanced age • High comorbidity burden ‘Charlson index 3 points higher in Ceiling of Care patients’ • Multiple comorbidities • Frailty: Assessed using the Rockwood Clinical Frailty Scale	• Higher mortality • Increased severe pneumonia in subjects with a Ceiling of Care (RR ranging from 1.2 to 1.4). • More complications among subjects with a Ceiling of Care • More antibiotic use in subjects with a ceiling of care • No difference in receiving other treatments

Author Pallarès et al. [27] Country Spain and the United Kingdom Setting Inpatient wards of general hospitals	To develop and validate a clinical prediction model to predict Ceiling of Care decisions using information readily available at the point of hospital admission	Study design Prediction model development and validation study using logistic regression with internal and external validation Population Hospitalised adults with proven COVID‐19 (*n* = 6207) Term used in the article Therapeutic Ceiling of Care	• Clinically appropriate care ceiling • Based on patients’ clinical profile • Resource‐contingent decision‐making • Anticipatory planning • Shared, multiparty decision‐making	• Pandemic context • Resource constraints • Advanced age • Multiple comorbidities	• Higher mortality • Increased complications in subjects with a therapeutic Ceiling of Care • Limited ICU access • No IMV, patients with ceiling received minimal mechanical ventilation

Author Piers et al. [32] Country Multiple European countries (11 countries across 4 regions) Setting AGUs in hospitals	To describe the goals‐of‐care discussions and treatment‐limitation decision practices in hospitalised older people across European AGUs.	Study design Cross‐sectional international multicentre study Population Older adults (≥ 75 years) (*n* = 590) Term used in the article Treatment‐limitation decision	• Actionable clinical order • Dynamic, ongoing and reversible planning • Linked to goals of care • Benefit–burden evaluation	• Acute health crisis requiring hospitalisation • Frailty: ‘The median premorbid CFS was six, interquartile range (IQR) [5–7], with 77% of patients having CFS of five or higher and 32% CFS of seven or higher’ • Multiple comorbidities • Advanced age	• Reduced nonbeneficial interventions • Goal‐concordant care delivery • Shorter hospital stays • Reduced invasive procedures • Improved quality of dying • Alignment with patient preferences

Author Slack and Rose [28] Country United Kingdom Setting Older Person’s Mental Health Services	To improve the recording of Ceiling of Care decisions in electronic notes to provide guidance for on‐call trainees when reviewing deteriorating patients out of hours	Study design Quality‐improvement project (PDSA cycles) Population Older persons with mental health conditions, particularly dementia, frail with medical comorbidities (First cycle, *n* = 14 Second cycle, *n* = 17) Term in used in the article Ceiling of Care	• Formal documentation • Involve the patient and family • Dynamic, ongoing and reversible Planning • Anticipatory planning • Deterioration‐responsive planning	• Advanced dementia • Frailty • Multiple comorbidities • Risk of deterioration	• Reduced unnecessary transfers to the hospital • Guides out‐of‐hours decision‐making • Improved guidance • Clearer documentation • Reduced on‐call burden • Improved compliance among doctors: ‘80% (4/5) fully compliant with new criteria and then 71% (5/7) in successive cycles’

Author Straw et al. [29] Country United Kingdom Setting Acute inpatient wards	To report Ceiling of Care and cardiopulmonary resuscitation decisions and their association with demographic and clinical characteristics and outcomes during the COVID‐19 pandemic.	Study design Retrospective observational study Population Hospitalised adults with proven COVID‐19 (*n* = 599) Term used in the article Ceiling of Care	• Anticipatory planning • Formal documentation • Benefit–burden evaluation • Shared, multiparty decision‐making	• Frailty: The median premorbid CFS was six, interquartile range (IQR) [5–7], with 77% of patients having CFS of five or higher and 32% CFS of seven or higher • Multiple comorbidities • Advanced age	• Avoidance of unwanted intensive treatment • Appropriate resource utilisation • Benefit‐focused care delivery • Facilitated timely decision‐making • Guided treatment delivery

Author Vranas et al. [33] Country United States Setting EDs	To evaluate how POLST form completion and treatment limitations influence the intensity of treatment among patients presenting to the ED.	Study design Retrospective cohort study Population Adult ED patients (*n* = 26,128) Term used in the article POLST	• Intended for patients with advanced illness/frailty approaching • Formal documentation	• Advanced illness • Frailty • Approaching the end of life • Need for treatment preference documentation • Advanced age • Multiple comorbidities	• Reduced ICU admission for treatment limitations: ‘treatment limitations on POLST forms were associated with lower odds of ICU admission (OR = 0.31; 95% CI 0.16–0.61)’ • Shorter hospital length of stay • Alignment with goals of care • Reduced time to discharge: ‘POLST was associated with decreased hospital and ICU length of stay compared to patients without POLST forms (hospital length of stay HR 1.14 [95% CI 1.06 to 1.23] and ICU length of stay HR 1.52 [95% CI 1.12 to 2.07])’ • Prevention of unwanted interventions

Author [25] Country Multiple countries across Europe (primarily France, Spain, Italy, United Kingdom) Setting EDs	To determine whether patient gender influences emergency physicians’ decisions regarding the recommendation for tracheal intubation in critically ill patients	Study design Survey‐based study Population Emergency physicians (*n* = 3423) Term used in the article Ceiling of Care	• Clinically appropriate care ceiling • Shared, multiparty decision‐making • Considers multiple factors • Patient values elicitation • Influenced by patient functional status	• Acute deterioration • Healthcare provider beliefs and biases • Gender‐based implicit bias	• Treatment‐limitation implementation • Alignment with patient goals • Gender‐based disparity in recommendations

Author Walzl et al. [7] Country United Kingdom Setting EDs	To determine the factors that influence ceiling of treatment institution in the ED.	Study design Phenomenological qualitative study semistructured interviews Population Emergency medicine consultants (*n* = 15) Term used in the article Ceiling of Treatment	• Actionable clinical order • Value‐concordant decision‐making • Alignment with patient values • Benefit–burden evaluation • Formal documentation • Stepwise process	• Advanced age • Multiple comorbidities • Patient’s lack of capacity • Low prevalence of advance directives • Trigger of illness severity	• Futility avoidance • Family and patient involvement

Author Walzl et al. [58] Country Multiple countries Setting EDs	To identify factors that influence decisions to limit treatment in the Emergency Department through a systematic search of the available literature.	Study design Systematic review Population Critically ill patients in ED *(n* = 2420) Term used in the article Treatment‐limiting decisions	• Associated with functional limitation • Patient values elicitation • Communication‐driven decision‐making	• Advanced age • Associated with chronic disease • Acute deterioration • Multiple comorbidities • Lack of patient capacity	• Improved patient and family experience • Reduced unnecessary hospital burden • Colleague consultation involvement • Variability in decision‐making between physicians and units

*Note:* ReSPECT, Recommended Summary Plan for Emergency Care and Treatment; SAMBA, the Society for Acute Medicine Benchmarking Audit.

Abbreviations: ACP, advanced care plan; AGU, acute geriatric units; CFS, Clinical Frailty Scale; DNACPR, Do Not Attempt Cardiopulmonary Resuscitation; ED, emergency department; HECT, hospital emergency care team; HR, hazard ratio; ICU, intensive care unit; IMV, invasive mechanical ventilation; POLST, physician orders for life‐sustaining treatment.

### 2.5. Data Synthesis and Analysis

Inductive thematic analysis, following Braun and Clarke’s six‐phase method [20], was undertaken within Walker and Avant’s method to analyse the extracted data. The first author (HAQ) read all included studies repeatedly, then systematically extracted all identified uses and definitions of Ceiling of Care concept. Subsequently, two authors (HAQ and HAD) collaboratively coded the extracted data line by line, using discussion and consensus to determine defining attributes (the essential characteristics distinguishing the concepts), antecedents (preceding conditions) and consequences (outcomes) [17]. Initial codes were collated and grouped into potential themes. These themes were iteratively reviewed and refined to ensure internal coherence and alignment with Walker and Avant’s analytical categories. Any discrepancies or ambiguities in coding or theme development were resolved through discussion with a third author (YS).

Walker and Avant’s [17] eight‐step method was used to guide data analysis and development of the concept of Ceiling of Care (Table 2). Step 1 (selection of the concept, Ceiling of Care) and Step 2 (determining the aims and purpose of the analysis) were completed during the study design phase; analytic work commenced with Step 3. Guided by the framework, the team systematically identified all uses of the term across the included studies, determined defining attributes that distinguish Ceiling of Care from related concepts, and articulated antecedents and consequences. Subsequently, to further define conceptual boundaries and facilitate operationalisation, the team developed a model case, as well as borderline, related and contrary cases and identified empirical referents to support future application of the concept in clinical practice and research.

## 3. Study Findings

### 3.1. Characteristics of Included Studies

This concept analysis included 19 studies published between 2012 and 2025. Studies were conducted in the United Kingdom (*n* = 6), through multicountry collaborations (*n* = 6), the United States (*n* = 4), France (*n* = 1), Spain (*n* = 1) and Australia (*n* = 1). Designs included quantitative studies (*n* = 11), qualitative studies (*n* = 3), reviews (*n* = 2), quality‐improvement projects (*n* = 2) and a case report (*n* = 1). Across the studies, the total sample comprised 51,204 hospitalised patients and 3438 clinicians. Only one study included nurses within a combined sample of registered nurses and social workers [21], highlighting the limited representation of nursing perspectives in the conceptualisation of Ceiling of Care. Emergency departments were the most common setting (*n* = 10), followed by inpatient wards (*n* = 7), combined emergency and inpatient settings (*n* = 1) and an acute geriatric unit (*n* = 1). Study populations included older adults and patients with COVID‐19, advanced cancer, end‐stage kidney disease or dementia, commonly characterised by frailty, comorbidity, functional impairment or acute deterioration. Terminology was heterogeneous and included Ceiling of Care or treatment, advance care planning, treatment‐limiting decisions, TELP, ReSPECT and POLST. The characteristics and key findings of the included studies are presented in Table 3.

### 3.2. Concept Analysis Result

#### 3.2.1. Use of the Concept of Ceiling of Care

Following Walker and Avant’s [17] method, an analysis of the use of the concept of Ceiling of Care in the healthcare literature was conducted. Ceiling of Care does not appear as a standalone term in general dictionaries. The term combines two components: ‘care’ and ‘ceiling’. In the Merriam‐Webster dictionary, ‘care’ functions as both a noun, referring to responsibility for or attention to health, well‐being and safety, and a verb, meaning to feel interest or concern and to provide care [22]. ‘Ceiling’ functions as a noun and is defined as an upper, usually prescribed, limit or the top of things [23].

The earliest identified use of this concept in literature dates to 2006, when noninvasive ventilation was described as ‘a ceiling of treatment’ for older adult patients with acute exacerbation of chronic obstructive pulmonary disease (COPD) for whom endotracheal intubation was deemed inappropriate [10]. Since then, the term has evolved to describe the maximum level of medical intervention considered appropriate for an individual patient, determined by clinical status, anticipated benefit and patient wishes [7, 24, 25]. However, the literature demonstrates a substantial variation in how this concept is defined and applied. In some cases, the concept is applied without definition altogether, with operational criteria specified for who may authorise a decision and how it is recorded, but no account of the construct itself [26]. For instance, Walzl et al. [7] identified three operational levels of Ceiling of Care: full escalation, limited escalation and continuing current care, while maintaining the option to initiate palliative care, whereas others [27–29] have used the term more broadly to describe any treatment‐limiting or escalation decision. The included studies in the review indicated that this therapeutic threshold was expressed through related terminologies, including advance care planning, treatment‐limiting decisions, TELP, ReSPECT and POLST. Although these approaches emphasise different aspects of treatment decision‐making, they share an interest in establishing therapeutic boundaries. Definitions identified across the included studies are provided in Supporting File 1.

Ceiling of Care provides a framework for guiding ethical treatment decisions, explicitly linking clinical judgement to the four principles of biomedical ethics: autonomy, beneficence, nonmaleficence and justice [30, 31]. It operationalises autonomy by integrating patient values and preferences into escalation decisions [25], while beneficence and nonmaleficence are balanced through an assessment of potential benefits against burdens [29]. Justice considerations emerge when ceiling determinations involve resource allocation, raising questions about equitable access to intensive therapies [24, 27].

Beyond ethics, Ceiling of Care has also been discussed in relation to legal and professional responsibilities, particularly in the context of best‐interest decision‐making when patients lack capacity. In such cases, it provides a framework for clinicians to make defensible and transparent treatment decisions consistent with their duty of care [31]. These decisions are often recorded in structured documentation tools such as ReSPECT forms or local goals‐of‐care plans, reinforcing accountability and continuity of care [32, 33].

From a practical perspective, Ceiling of Care functions as a communication and coordination tool across clinical teams. It guides decisions regarding admission, transfer and escalation, for example, determining when ICU transfer is not appropriate, and provides clarity for staff managing deterioration or end‐of‐life care [28, 29]. In this sense, Ceiling of Care serves not only as an ethical and legal construct but also as an operational instrument that translates complex treatment boundaries into actionable clinical guidance. Collectively, these dimensions demonstrate the concept’s multifaceted nature, encompassing moral, procedural and communicative functions that underpin its defining attributes in acute care practice.

#### 3.2.2. Determining the Defining Attributes

**FIGURE 2:**
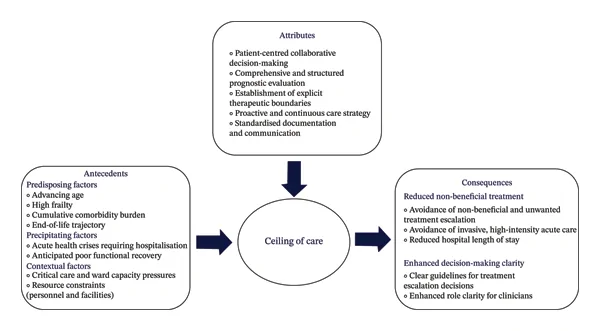
Conceptual model of ceiling of care in acute care settings.

According to Walker and Avant [17], defining attributes represent the characteristics most frequently associated with a concept, serving to distinguish it from related concepts. Following a comprehensive review of the literature, a systematic record of features frequently associated with Ceiling of Care concept was created (Figure 2). Recurring characteristics were identified and clustered into attributes that collectively capture the essence of Ceiling of Care. Five defining attributes emerged from this analysis: (1) patient‐centred collaborative decision‐making; (2) comprehensive and structured prognostic evaluation; (3) establishment of explicit therapeutic boundaries; (4) proactive and continuous care strategy and (5) standardised documentation and communication.

##### 3.2.2.1. Attribute 1: Patient‐Centred Collaborative Decision‐Making

Ceiling of Care is defined by a decision‐making process that seeks to align therapeutic limits with the patient’s known values, goals and preferences [29]. This attribute functions as a negotiation between clinical expertise and personal autonomy: Healthcare teams define the range of medically appropriate interventions based on prognosis and clinical assessment, while patients contextualise these options within their comfort and quality‐of‐life expectations [7, 25, 31]. Collaboration is described variably, extending in some accounts beyond the responsible clinician to include nurses and social workers in the discussion [21], and in some cases involving procedural safeguards between clinicians, like requiring approval from two senior doctors from different wards [26]. This encompasses both process and outcome, wherein patients and families actively engage in discussions, and the final decision reflects the patient’s wishes [26, 28, 29]. In clinical practice, patients frequently lack capacity at the time of deterioration; Misselbrook et al. [31] reported capacity was absent in 45% of cases. In such circumstances, next‐of‐kin and surrogate decision‐makers represent patient interests and known wishes, ensuring that the collaborative process continues even when patients cannot participate directly [27, 29]. This attribute was consistently supported across the literature, although the extent of patient and family involvement varied, with some decisions remaining physician‐led.

##### 3.2.2.2. Attribute 2: Comprehensive and Structured Prognostic Evaluation

Ceiling of Care decisions are grounded in a comprehensive assessment of each patient’s clinical situation rather than the uniform application of standardised protocols. This assessment encompasses the evaluation of prognosis, existing comorbidities, current disease trajectory, functional status, and the balance between potential benefits and burdens of treatment [25, 27]. Importantly, this attribute is distinct from the assessment of acute severity; patients assigned a restrictive ceiling may present with less severe acute symptoms but possess higher background vulnerability and lower physiological reserve [24, 26, 27]. The literature characterises this assessment not as a subjective judgement but as a structured evaluation, often operationalised through validated prognostic instruments such as the Clinical Frailty Scale [26, 29] or the Supportive and Palliative Care Indicators Tool [31]. This individualised approach ensures that the established ceiling reflects realistic clinical expectations and anticipated outcomes for each patient [7], recognising that identical interventions may be appropriate for one patient but inappropriate for another depending on individual circumstances and likelihood of benefit. Overall, the literature consistently supported comprehensive, multifactorial prognostic evaluation, although evidence for a uniformly structured assessment process was more limited and based mainly on the use of selected prognostic tools.

##### 3.2.2.3. Attribute 3: Establishment of Explicit Therapeutic Boundaries

The primary defining attribute of a Ceiling of Care is the establishment of a defined maximum treatment limit. This attribute conceptualises the ceiling as a clear upper boundary of therapeutic intensity, rather than a binary choice between full treatment and palliation [7]. It represents the maximum therapeutic effort to be offered or the highest appropriate level of care for a patient [24, 27]. This graduated structure is evident in practice: Walzl et al. [7] described three levels of Ceiling of Care, comprising full escalation, limited escalation and continuing current care, while Coisy et al. [26] similarly graded treatment limitation across three levels as low, medium or high. For example, a defined ceiling may permit advanced respiratory support while simultaneously precluding intubation or intensive care admission [29]. The explicit nature of this limit clarifies for healthcare providers, patients and families which treatments are considered appropriate, thereby preventing both under‐ and overtreatment in clinical practice. This attribute is the most directly and consistently supported in the literature, with multiple studies defining Ceiling of Care through an explicit maximum level of treatment or escalation.

##### 3.2.2.4. Attribute 4: Proactive and Continuous Care Strategy

Ceiling of Care functions as an anticipatory plan, distinguishing it from reactive decisions made during acute physiological collapse. Three studies characterise this as a pre‐emptive process designed to guide management before a crisis occurs, shifting decision‐making away from distressing acute deterioration emergencies during which patients may lack capacity [21, 29, 31]. These plans are frequently established at hospital admission, utilising available clinical data to stratify risk and set limits early in the patient’s journey [24, 27]. Limits of treatment may also be set at emergency department arrival, where the level determined has been shown to shape the remainder of the admission [26]. Equally important, Ceiling of Care is dynamic rather than static; decisions are reviewed and updated as circumstances change, whether through clinical improvement, deterioration or evolving patient preferences [7, 25, 31]. The level determined in one setting may be revised following transfer to another [26]. Thus, Ceiling of Care represents a continuous strategy, ensuring that therapeutic limits remain current throughout the care trajectory. Overall, anticipatory planning was consistently supported across the literature, while evidence for continuous review was also present but reflected a variation in when and how decisions were reassessed.

##### 3.2.2.5. Attribute 5: Standardised Documentation and Communication

The final defining attribute of Ceiling of Care is standardised documentation and communication, which operationalises the clinical decision into a retrievable medical order that applies across different care settings. The literature distinguishes this attribute from ad‐hoc clinical notes, requiring the use of recognised, portable frameworks such as the ReSPECT [31] or POLST [33] to ensure that therapeutic limits are visible to clinicians who have no prior relationship with the patient [28, 29]. Documented decisions are translated into actionable clinical orders that guide care delivery [24, 33]. Where entry in the record is a procedural requirement rather than left to discretion, the recorded level demonstrably governs subsequent management [26]. This distinguishes the ceiling from an advance directive, which records preferences that require interpretation rather than specifying a level of intervention that can be applied directly [21]. Decisions must be consistently documented in a designated, accessible location to ensure visibility and continuity of care, particularly in settings where electronic records are used [31]. When properly documented, Ceiling of Care decisions significantly influence clinical practice, particularly during emergencies or when new clinicians are involved [7]. Overall, formal documentation and communication were consistently supported, although the formats and degree of standardisation varied across frameworks and healthcare settings.

#### 3.2.3. Model Case

Identifying model cases is the fifth step in Walker and Avant’s [17] method for concept analysis. A model case is a case that contains all defining attributes [17]. A model case may be sourced from the literature, derived from personal experience, or constructed, and should serve as an exemplar containing all the defining attributes of the concept [17]. The following case was constructed by the authors to illustrate how the five defining attributes of Ceiling of Care function together in practice.

Mr Stuart Johns, a 78 year‐old with advanced COPD, heart failure, chronic kidney disease and declining functional status, is admitted following an exacerbation. Dr Jonas Samuels reviews his history and disease trajectory, concluding that intensive care is unlikely to offer meaningful benefit. He discusses the available options with Mr Johns, who prioritises the quality of life and comfort and expresses a wish to avoid ICU admission and long‐term ventilation. His daughter, who holds power of attorney, affirms these wishes. Following collaborative discussion involving Mr Johns, his daughter and his doctor, a Ceiling of Care plan is established: ward‐based care with noninvasive ventilation as maximum respiratory support, transitioning to comfort measures if this proves insufficient. A DNR decision is documented as part of the holistic plan using the hospital’s standardised form, entered into the electronic medical record with an alert and translated into specific clinical orders. Three days later, Mr Johns deteriorated overnight. The on‐call physician, unfamiliar with Mr Johns’ case, accesses the documented Ceiling of Care for guidance. Noninvasive ventilation is initiated, but referral to the ICU is not pursued, consistent with the documented plan. One week later, following improvement, Dr Samuels reassesses Ceiling of Care with Mr Johns and his daughter, confirms that preferences remain unchanged and updates the documentation accordingly.

This case demonstrates all five defining attributes of Ceiling of Care. Mr Johns’s case illustrates how Ceiling of Care is established through individualised assessment, collaborative decision‐making aligned with patient values, anticipatory planning and standardised documentation, resulting in a specified maximum treatment limit that guides clinical care. The case further demonstrates the dynamic nature of Ceiling of Care through reassessment following clinical change.

#### 3.2.4. Additional Cases

Identification of additional cases, including borderline, related and contrary cases, is the sixth step in Walker and Avant’s [17] method. A borderline case contains most but not all defining attributes; a related case shares similarities but differs in important ways; and a contrary case lacks most or all defining attributes, thereby illustrating what the concept is not. The authors have constructed the following cases.

##### 3.2.4.1. Borderline Case

Mr Yasin, an 82 year‐old patient with advanced dementia and recurrent aspiration pneumonia, is admitted to the hospital. The medical team assesses his prognosis and functional status and concludes that intensive care would not be beneficial. His son, who holds power of attorney, agrees that ward‐based care with antibiotics represents the maximum appropriate treatment. However, this decision is not documented in the medical record. When Mr Yasin deteriorates overnight, the on‐call physician cannot locate the decision and must re‐initiate discussions with the family. This case demonstrates most of the defining attributes: the establishment of explicit therapeutic boundaries; a comprehensive, structured prognostic evaluation; patient‐centred, collaborative decision‐making; and a proactive, anticipatory care strategy. However, it lacks standardised documentation and communication, thereby compromising clinical utility.

##### 3.2.4.2. Related Case

Mrs Nancy, a 70 year‐old patient with metastatic lung cancer, is admitted with pneumonia. She states that she would not want cardiopulmonary resuscitation, and a DNR order is documented. However, no broader discussion of other treatment limitations occurs. No maximum treatment level is defined, and no anticipatory plan for deterioration is established. When Mrs Nancy later deteriorates, the team is uncertain whether to escalate care to the ICU. This represents a related case because DNR shares features with Ceiling of Care; both involve treatment limitation and documentation. However, DNR addresses only cardiac arrest, whereas Ceiling of Care establishes a comprehensive maximum treatment boundary that guides management prior to arrest.

##### 3.2.4.3. Contrary Case

Mr Ahmad, a 65‐year‐old man with no significant medical history, is admitted with pneumonia. No discussion regarding treatment limitations occurs, and no assessment of his values or goals of care is undertaken. On Day 3, Mr Ahmad unexpectedly deteriorates. The on‐call team, without documented guidance, intubates him and transfers him to the ICU. His family later expressed that he had previously stated he would never want to be ‘kept alive on machines’. This case lacks all five defining attributes. Treatment decisions are made reactively, in a crisis, in accordance with default medical protocols, without patient input or prior planning, thereby demonstrating the absence of Ceiling of Care.

#### 3.2.5. Identify Antecedents and Consequences

##### 3.2.5.1. Antecedents

Walker and Avant [17] define antecedents as events or incidents that must occur prior to the occurrence of the concept. The antecedents derived from the literature were categorised into three groups: predisposing factors, precipitating factors and contextual factors relating to system‐level constraints.

###### 3.2.5.1.1. Predisposing Factors

The literature consistently identifies limited physiological reserve as a key factor influencing decisions to limit treatment escalation. Physiological reserve reflects an individual’s capacity to tolerate acute illness and recover from invasive interventions and is commonly operationalised through indicators such as advanced age [7, 24, 26, 27, 29], high frailty [24, 26, 31, 33], cumulative comorbidity burden [7, 29, 33] and a diagnosis carrying a risk of sudden deterioration or loss of capacity, such as advanced or incurable cancer [21]. Together, these factors are strongly associated with decisions to limit treatment intensity, as patients with reduced reserve are less likely to benefit from aggressive interventions. Additionally, the recognition of an end‐of‐life trajectory further informs treatment planning by guiding a transition toward anticipatory, comfort‐focused and goal‐concordant care. [33].

###### 3.2.5.1.2. Precipitating Factors

Acute health crises requiring hospitalisation commonly precipitate assessment of Ceiling of Care [24]. Even when patients are admitted without an established Ceiling of Care, acute deterioration following admission may prompt its initiation [28, 31, 33]. Importantly, the trigger is not simply physiological decline; rather, it is the clinician’s judgement that the patient is unlikely to benefit from intensive treatment [7, 29]. Anticipated poor functional recovery also precipitates such decisions, with management guided by the likelihood of benefit [28, 29, 33].

###### 3.2.5.1.3. Contextual System Constraints

Contextual antecedents reflect structural health system conditions that shape the feasibility and framing of Ceiling of Care, beyond patient‐level factors alone. Periods of heightened service demand, most notably during the COVID‐19 pandemic, exposed how capacity pressures and resource scarcity function as antecedent conditions, prompting earlier and more explicit treatment‐limitation decisions in acute care settings [24, 29]. In these contexts, Ceiling of Care was not merely a clinical judgement but was embedded within system‐level imperatives to prioritise limited critical care resources [27]. Importantly, the influence of system constraints is not confined to pandemic conditions. Even in routine practice, factors such as intensive care unit occupancy, bed availability and staffing capacity shape escalation thresholds and the timing of treatment‐limitation discussions [7]. Constraints also arise within the workforce rather than in physical resources. Emergency department overcrowding and workload [26], clinician reluctance and insufficient time or training [21], and the discretionary rather than required status of the planning process itself [21] each condition whether limits are set at all. These findings indicate that Ceiling of Care is situated within broader organisational and resource contexts, positioning system constraints as a distinct antecedent domain rather than a secondary consideration.

#### 3.2.6. Consequences

Consequences are events or outcomes that occur as a result of the concept [17]. The consequences derived from the literature were categorised into two groups: reduced nonbeneficial treatment and enhanced decision‐making clarity.

##### 3.2.6.1. Reduced Nonbeneficial Treatment

Implementation of Ceiling of Care consistently reduces escalation to unwanted treatments and high‐intensity interventions. Studies demonstrate that Ceiling of Care leads to fewer ICU admissions and reduced use of invasive ventilation [7, 24, 29]. Vranas et al. [33] found that among patients with comfort‐measures‐only orders, only 1.2% were admitted to the ICU, and none received cardiopulmonary resuscitation. Additionally, documented decisions help avoid unnecessary transfers to acute hospitals outside regular hours, reducing patients’ financial burden [28]. Ceiling of Care is also associated with reduced hospital utilisation, including shorter lengths of stay and earlier discharge [29, 33]. Collectively, these outcomes reflect the avoidance of futile treatment escalation, aligning care with anticipated benefit and patient goals [7]. Ceilings also support the avoidance of care disproportionate to the clinical condition [26]. Regarding clinical outcomes, Pallares et al. [24] found that studies on patients with a restrictive ceiling during the COVID‐19 pandemic had a mortality rate more than four times higher than that of patients without a restrictive ceiling, with a higher incidence of pneumonia and increased antibiotic use; however, this association likely reflects underlying patient vulnerability and the pandemic context rather than adverse effects of Ceiling of Care itself.

##### 3.2.6.2. Enhanced Decision‐Making Clarity

Ceiling of Care has important implications for clinical decision‐making and care coordination. Documented ceilings provide clear escalation guidance, supporting timely, preference‐concordant decisions, particularly for junior and out‐of‐hours clinicians [31]. Such documentation provides critical support to out‐of‐hours doctors, reducing pressure to make high‐stakes decisions without adequate information [28]. Misselbrook et al. [31] reported that junior doctors experienced reduced anxiety in an environment that fostered open communication. Clarity established early appears to carry forward: The level set at presentation has been associated with placement in a setting matched to it, whether ward, intensive care, palliative care unit or home [26]. Establishing a ceiling also provides an explicit point at which palliative involvement can be considered without displacing active treatment [7, 26]. Furthermore, judicious ceilings prevent excessive allocation of scarce resources to futile treatments [7].

#### 3.2.7. Empirical Referents

According to Walker and Avant [17], the final step of concept analysis involves identifying empirical referents, which are observable categories in real‐world practice that signify the existence of a concept by reflecting its defining attributes. Structured care‐planning frameworks used in clinical practice provide measurable examples of such referents. This analysis indicates that the ReSPECT framework functions as a robust empirical referent for the concept of Ceiling of Care. ReSPECT is a structured process for emergency care and treatment planning that supports collaborative decision‐making regarding appropriate emergency interventions and records consensus clinical recommendations for situations when an individual cannot communicate their preferences [31].

The ReSPECT process begins with a conversation between the healthcare professional and the patient, establishing a shared understanding of the person’s current health status, diagnoses and relevant circumstances [34]. This conversation explores what matters most to the patient, capturing their values on a scale from ‘living as long as possible matters most to me’ to ‘quality of life and comfort matters most to me’, as well as what they most value and what they most fear or wish to avoid. When the patient lacks capacity, the process involves family members or legal welfare proxies to ensure that known wishes and best interests guide the discussion. Based on this shared understanding, clinicians document clinical recommendations on a spectrum from ‘prioritise extending life’ through ‘balance extending life with comfort and valued outcomes’ to ‘prioritise comfort’, specifying which emergency interventions are clinically appropriate or inappropriate, including a clear recommendation regarding CPR. The plan is dated, signed by the responsible clinician, and includes explicit provision for review when circumstances change. The finalised ReSPECT form serves as an identifiable, portable record that accompanies the patient, accessible to any clinician involved in their care, ensuring that established recommendations guide treatment during acute deterioration.

Through this process, ReSPECT demonstrates all five defining attributes of Ceiling of Care within a single, integrated framework, providing a tangible illustration of the concept in clinical practice.

#### 3.2.8. Proposed Definition

Based on the defining attributes, antecedents and consequences identified through this analysis, we propose the following definition: Ceiling of Care is an anticipatory, documented clinical decision‐making approach that establishes the maximum appropriate level of treatment escalation for an individual patient, based on clinical assessment, anticipated benefit and patient values, guiding ongoing care and escalation decisions in acute settings. This definition deliberately emphasises anticipatory rather than reactive planning, integrates clinical and patient‐centred elements, and frames Ceiling of Care as ongoing guidance rather than a discrete decision, thereby addressing the conceptual confusion that has limited consistent application of practice in acute care settings. Figure 2 integrates the three categories of antecedents, five defining attributes and two categories of consequences into a conceptual model of Ceiling of Care in acute care settings. A visual comparison of how Ceiling of Care differs from traditional orders is summarised in Table 1.

## 4. Discussion

This concept analysis extends current understanding of treatment‐limitation frameworks by reconceptualising Ceiling of Care as an anticipatory, ethically grounded approach to decision‐making rather than a reactive administrative label. This distinction is not merely semantic: It challenges prevailing assumptions that escalation limits are crisis‐driven responses, positioning Ceiling of Care instead as a proactive framework for aligning medical judgement with patient values. Following Walker and Avant’s [17] method, we identified five defining attributes, three antecedents and two consequences, which together formed the basis for the proposed definition. This definition clarifies the conceptual ambiguity surrounding the understanding and application of Ceiling of Care in clinical practice.

A central contribution of this analysis is the distinction between Ceiling of Care and related concepts frequently conflated in practice, particularly DNR orders. DNR orders are specific to cardiac arrest and focus on withholding intervention [35]. In contrast, Ceiling of Care functions as a broader framework that establishes explicit therapeutic boundaries and proactive care strategies to manage acute deterioration before cardiac arrest occurs.

Failing to distinguish these concepts reveals a critical gap in treatment‐limitation frameworks. DNR status is often interpreted variably [36, 37] and may unintentionally signal broader limitations on treatment options [38, 39]. Evidence suggests this misinterpretation can lead to the inappropriate withholding of active therapies, such as withholding blood products, or not administering antibiotics [40], even when patients could benefit from a scope of care [37, 41, 42]. These are not isolated clinical errors but predictable consequences of conflating resuscitation status with treatment intensity. By defining treatment boundaries independently of resuscitation status, Ceiling of Care provides more explicit guidance for ongoing clinical management.

Ceiling of Care is conceptually distinct from treatment‐limiting decisions, which are typically reactive responses to clinical deterioration, treatment failure or the development of additional organ dysfunction [43, 44]. While those decisions focus on withholding discrete therapies [45], Ceiling of Care is an anticipatory framework that establishes treatment boundaries before a crisis occurs, shifting the care from crisis‐driven rationing to proactive, values‐based care planning. This difference matters because reactive limitations happen when patients cannot participate in decisions, families are distressed, and clinicians face moral dilemmas under time pressure. Viewing ceiling decisions as anticipatory rather than reactive challenges the idea that treatment limits are a crisis response. Instead, they are an ethical planning process that respects patient autonomy and eases clinician burden.

Three identified attributes, comprehensive prognostic evaluation, proactive care strategy and patient‐centred, collaborative decision‐making, position Ceiling of Care as a strategy established before a crisis. Ceiling of Care also challenges the binary choice between full escalation and palliative care. Instead, it enables a graduated spectrum of interventions, ranging from ward‐based care to advanced multiorgan support, tailored to the individual [7]. This approach shifts how boundaries are set, potentially reducing over‐ and undertreatment while facilitating early palliative care involvement for symptom management [46].

As a patient‐centred framework, Ceiling of Care integrates the patient’s wishes and values in acute care. The literature indicates that clinicians often act inconsistently with expressed preferences, and families frequently struggle to make decisions once a patient loses capacity [47]. Furthermore, insufficient exploration of patients’ values can lead to misaligned expectations regarding survival benefits [48]. Ceiling of Care addresses these challenges by establishing treatment boundaries while patients retain capacity, creating a foundation for values‐aligned care. While further empirical validation is required to determine if this framework improves decision quality and family confidence, preliminary evidence suggests that structured advance care planning reduces family distress [49], warranting further investigation into Ceiling of Care implementation.

### 4.1. Implications for Nursing Management

Given that nurses are central to care coordination within acute care settings, clarifying this concept and its implications for nursing practice is essential for supporting consistent, ethically sound, patient‐centred decision‐making. The conceptual clarity achieved through this analysis provides a roadmap for three fundamental practice changes: the development of standardised documentation with health record integration; the implementation of targeted clinical education programs; and the establishment of systematic protocols for initiating and updating ceiling discussions, moving Ceiling of Care from a theoretical construct into a functional clinical tool. In practice, the five defining attributes may be operationalised through structured care‐planning processes, such as ReSPECT, which integrate values‐based discussion, explicit therapeutic boundaries, accessible documentation and planned review.

At the organisational level, the first priority is the development of standardised Ceiling of Care documents tailored to specific cultural and policy contexts, such as ReSPECT. Unlike isolated DNR orders, these documents provide a nuanced outline of varying intervention levels and must be seamlessly integrated into electronic health records to ensure they are accessible the moment a patient’s condition worsens. However, structural tools alone are insufficient; they need to be supported by a robust clinical education program. This training needs to clarify beyond conceptual distinctions between Ceiling of Care and related concepts. It also needs to build essential clinician competencies in prognostic assessment, value elicitation and the nuances of collaborative decision‐making. To ensure these skills are applied consistently, institutions should mandate Ceiling of Care discussions at defined ‘trigger points’, such as admission with advanced illness, signs of clinical deterioration or key care transitions, supported by clear documentation standards across all multidisciplinary teams.

While the impact of these shifts on patient‐centred outcomes still requires formal empirical evaluation, the framework provides a basis for context‐sensitive implementation accompanied by ongoing assessment. Clear documentation of treatment escalation addresses a significant vulnerability in current acute care: the lack of accessible guidance during a crisis. When clinical teams encounter deterioration, particularly after‐hours or in the absence of senior clinicians, explicitly documented escalation limits provide the necessary certainty to inform immediate, high‐stakes decisions.

To truly maximise clinical utility, Ceiling of Care documentation must transition from a static note into a dynamic part of the clinical workflow. This requires that records are updated regularly as clinical situations evolve that a patient’s status is documented as it changes and that the language used remains standardised and consistently understood across professional groups. Ultimately, healthcare organisations need to prioritise electronic health record modifications that bring these decisions to the forefront, ensuring that Ceiling of Care limits are prominently displayed on patient summary screens and handover tools and within deterioration response protocols.

### 4.2. Implications for Future Research

This study emphasises conceptual analysis; however, implementation research is essential for translating conceptual clarity into practical application. Future research should identify factors influencing the implementation of Ceiling of Care across diverse acute care settings, with particular attention to conditions that facilitate successful adoption and inform strategies for broader expansion. Given the limited representation of nurses in the included studies, future research should examine acute care nurses’ understanding of Ceiling of Care, their participation in decision‐making, and the organisational and interprofessional factors that enable or constrain their involvement. Additionally, research must explore effective training approaches that build clinicians’ capability in prognostic assessment, value elicitation and collaborative decision‐making. Determining which educational interventions enhance confidence and competence in conducting Ceiling of Care discussions will be critical for workforce development.

Cultural and religious contexts require systematic investigation. None of the included studies addressed the influence of religion or cultural impacts on Ceiling of Care decision‐making, despite evidence that preferences vary significantly across populations. Cultural differences shape expectations regarding individual autonomy, family involvement and clinician guidance in treatment planning [50]. Future research should empirically test this conceptual model by examining whether clearly documented ceilings reduce treatment variability, moral distress and decisional conflict among clinicians and families. Additionally, exploring how organisational culture mediates the translation of conceptual clarity into clinical behaviour would help determine the model’s practical validity across settings.

### 4.3. Strengths and Limitations

A primary strength of this concept analysis lies in its rigorous and comprehensive search strategy, which incorporates database queries, manual reference checking, and AI‐assisted backward and forward citation searching. This multimodal approach ensured extensive coverage of the relevant literature. Furthermore, to address the common limitation of concept analyses being conducted by a single author [51], a team of three researchers collaborated during the search, screening and data extraction phases. This collaborative framework mitigated potential analytic bias and enhanced the overall rigour of the findings. The inclusion of the literature examining Ceiling of Care during the COVID‐19 pandemic represents a key strength of this review, as it provides unique insight into how the concept is operationalised under conditions of extreme clinical uncertainty and resource constraints, contexts that may not otherwise have been captured.

However, several limitations warrant consideration. First, only one included study involved nurses, within a combined sample of registered nurses and social workers, and findings were not reported separately by profession [21]. Consequently, nursing perspectives were substantially underrepresented, and the identified attributes may not fully reflect nurses’ experiences or their organisational role in Ceiling of Care decision‐making. Second, significant variability in how Ceiling of Care and related terms were defined, operationalised and documented across studies limited direct comparison and synthesis. Third, the majority of included studies were conducted in adult acute care settings within high‐income countries. Consequently, the findings may reflect specific legal frameworks, resource levels and organisational policies that differ substantially from those in low‐ and middle‐income settings, potentially limiting the global generalisability of the conceptualisation. Finally, restricting the inclusion to English‐language publications may have excluded relevant studies, thereby limiting the representation of culturally and contextually diverse perspectives on treatment‐escalation decision‐making.

## 5. Conclusions

This concept analysis establishes Ceiling of Care as a distinct and clinically significant framework within acute care, clearly distinguishing it from narrower constructs such as DNR orders and treatment‐limiting decisions. By applying Walker and Avant’s [17] methodology, we identified five defining attributes, along with their antecedents and consequences, to provide a robust conceptual foundation that addresses current practice gaps. Ceiling of Care is defined as an anticipatory clinical decision‐making approach that establishes the maximum appropriate level of treatment escalation for an individual patient. This approach is informed by comprehensive clinical assessment, anticipated benefit and the patient’s personal values, serving to guide ongoing care and escalation decisions within acute settings. Achieving conceptual clarity enables clinicians to set treatment boundaries proactively rather than reactively. The alignment of clinical care with patient values facilitates more ethically sound decision‐making and reduces the ambiguity often encountered during periods of rapid acute deterioration. By providing a shared language for treatment‐escalation boundaries, this analysis supports a vital paradigm shift in acute care: moving away from reactive crisis management toward structured, patient‐centred care planning.

## Author Contributions

Hussein Al‐Qarni and Yenna Salamonson were responsible for the study conception and design. Hussein Al‐Qarni, Yenna Salamonson and Hadwan Aldahmashi retrieved the data; Hussein Al‐Qarni, Yenna Salamonson and Hadwan Aldahmashi extracted the data, and Hussein Al‐Qarni and Hadwan Aldahmashi performed the data analysis. Hussein Al‐Qarni was responsible for drafting the manuscript. Yenna Salamonson, Della Maneze, Peta Drury and Luke Molloy made critical revisions to the paper for important intellectual content.

## Funding

Hussein Al‐Qarni is supported by a PhD scholarship from King Khalid University, Abha, Saudi Arabia. Open access publishing facilitated by the University of Wollongong, as part of the Wiley—University of Wollongong agreement via the Council of Australasian University Librarians.

## Ethics Statement

Ethical approval was not required for this paper.

## Conflicts of Interest

The authors declare no conflicts of interest.

## Supporting Information

Additional supporting information can be found online in the Supporting Information section.

## Supporting information


**Supporting Information 1** Supporting File 1: definitions of related terminologies and approaches used to establish therapeutic boundaries across the included studies.

**Supporting Information 2** PRISMA 2020 checklist.

## Data Availability

The data used to support the findings of this study are available from the corresponding author upon request.

## References

[1] Campling N. , Cummings A. , Myall M. et al., Escalation-Related Decision Making in Acute Deterioration: A Retrospective Case Note Review, BMJ Open. (2018) 8, no. 8, 10.1136/bmjopen-2018-022021. PMC6104759 30121604

[2] Dzeng E. and Curtis J. R. , Understanding Ethical Climate, Moral Distress, and Burnout: A Novel Tool and a Conceptual Framework, BMJ Quality and Safety. (2018) 27, no. 10, 766–770, 10.1136/bmjqs-2018-007905. PMC6540991 29669857

[3] Svantesson M. , Griffiths F. , White C. , Bassford C. , and Slowther A. , Ethical Conflicts During the Process of Deciding About ICU Admission: an Empirically Driven Ethical Analysis, Journal of Medical Ethics. (2021) 47, no. 12, 10.1136/medethics-2020-106672. PMC8639921 33402429

[4] DuMontier C. , Dale W. , Revette A. C. et al., Ethics of Overtreatment and Undertreatment in Older Adults With Cancer, BMC Medical Ethics. (2025) 26, no. 1, 10.1186/s12910-025-01255-9. PMC12291383 40707902

[5] Strand L. , Sandman L. , Tinghög G. , and Nedlund A.-C. , Withdrawing or Withholding Treatments in Health Care Rationing: an Interview Study on Ethical Views and Implications, BMC Medical Ethics. (2022) 23, no. 1, 10.1186/s12910-022-00805-9. PMC9233323 35751123

[6] Piscitello G. M. , Lyons P. G. , Koch V. G. , Parker W. F. , and Huber M. T. , Hospital Policy Variation in Addressing Decisions to Withhold and Withdraw Life-Sustaining Treatment, Chest. (2024) 165, no. 4, 950–958, 10.1016/j.chest.2023.12.028. PMC11026167 38184166

[7] Walzl N. , Jameson J. , Kinsella J. , and Lowe D. J. , Ceilings of Treatment: A Qualitative Study in the Emergency Department, BMC Emergency Medicine. (2019) 19, no. 1, 10.1186/s12873-019-0225-6. PMC6335704 30654741

[8] Davoudi N. , Nayeri N. D. , Zokaei M. S. , Fazeli N. , and Carspecken P. F. , Culture of Paternalism in the Emergency Department: A Critical Ethnographic Study, BMC Health Services Research. (2025) 25, no. 1, 10.1186/s12913-025-13282-8. PMC12344830 40797195

[9] Kaldjian L. C. , Clarifying Core Content of Goals of Care Discussions, Journal of General Internal Medicine. (2020) 35, no. 3, 913–915, 10.1007/s11606-019-05522-5. PMC7080909 31713035

[10] Balami J. , Packham S. , and Gosney M. , Non-Invasive Ventilation for Respiratory Failure due to Acute Exacerbations of Chronic Obstructive Pulmonary Disease in Older Patients, Age and Ageing. (2006) 35, no. 1, 75–79, 10.1093/ageing/afi211. 16364938

[11] Fan S.-Y. , Wang Y.-W. , and Lin I.-M. , Allow Natural Death Versus do-not-Resuscitate: Titles, Information Contents, Outcomes, and the Considerations Related to do-not-Resuscitate Decision, BMC Palliative Care. (2018) 17, no. 1, 10.1186/s12904-018-0367-4. PMC6180419 30305068

[12] Dias L. M. , Frutig M. d. A. , Bezerra M. R. , Barra W. F. , Castro L. , and Rego F. , Advance Care Planning and Goals of Care Discussion: Barriers From the Perspective of Medical Residents, International Journal of Environmental Research and Public Health. (2023) 20, no. 4, 10.3390/ijerph20043239. PMC9961136 36833934

[13] Cuevas-Østrem M. , Wisborg T. , Røise O. , Helseth E. , and Jeppesen E. , Decision-Making in Interhospital Transfer of Traumatic Brain Injury Patients: Exploring the Perspectives of Surgeons at General Hospitals and Neurosurgeons at Neurotrauma Centres, BMC Health Services Research. (2025) 25, no. 1, 10.1186/s12913-024-11968-z. PMC11817790 39934806

[14] Grossestreuer A. V. , Gaieski D. F. , Abella B. S. et al., Factors Associated With Post-Arrest Withdrawal of Life-Sustaining Therapy, Resuscitation. (2017) 110, 114–119, 10.1016/j.resuscitation.2016.10.021. PMC5541857 27840307

[15] Bakker J. , Huntink E. , Peters L. , Brugman I. , Ubbink D. , and Schoonhoven L. , Factors Influencing Shared Decision-Making on Hospital Wards as Perceived by Healthcare Professionals: A Qualitative Study, Applied Nursing Research. (2025) 81, 10.1016/j.apnr.2024.151892. 39864881

[16] Yamamoto K. , Yonekura Y. , and Nakayama K. , Healthcare Providers’ Perception of Advance Care Planning for Patients With Critical Illnesses in Acute-Care Hospitals: A Cross-Sectional Study, BMC Palliative Care. (2022) 21, no. 1, 10.1186/s12904-021-00900-5. PMC8742355 34996428

[17] Walker L. O. and Avant K. C. , Strategies for Theory Construction in Nursing, 2019, 6th edition, Pearson.

[18] Page M. J. , McKenzie J. E. , Bossuyt P. M. et al., The PRISMA 2020 Statement: An Updated Guideline for Reporting Systematic Reviews, BMJ. (2021) 372, 10.1136/bmj.n71. PMC8005924 33782057

[19] Wilson E. , Daniel M. , Rao A. et al., A Scoping Review of Distributed Cognition in Acute Care Clinical Decision-Making, Diagnosis. (2023) 10, no. 2, 68–88, 10.1515/dx-2022-0095. 36512433

[20] Braun V. and Clarke V. , Thematic Analysis: A Practical Guide, 2021, Sage.

[21] Jackson G. L. , Padilla B. I. , Schneider S. M. , and Kyte J. J. , Optimizing Advanced Care Planning in Hospitalized Patients With Advanced Cancers: A Quality Improvement Initiative, Journal of Doctoral Nursing Practice. (2019) 12, no. 2, 239–245, 10.1891/2380-9418.12.2.239. 32745036

[22] Merriam-Webster , Care, Merriam-Webster.com Dictionary, 2025, https://www.merriam-webster.com/dictionary/care.

[23] Merriam-Webster , Ceiling, Merriam-Webster.com Dictionary, 2025, https://www.merriam-webster.com/dictionary/ceiling.

[24] Pallares N. , Tebé C. , Abelenda-Alonso G. et al., Characteristics and Outcomes by Ceiling of Care of Subjects Hospitalized With COVID-19 During Four Waves of the Pandemic in a Metropolitan Area: A Multicenter Cohort Study, Infectious Disease and Therapy. (2023) 12, no. 1, 273–289, 10.1007/s40121-022-00705-w. PMC9736710 36495405

[25] Vromant A. , Alame K. , Cassard C. , Bloom B. , Miró-Andreu O. , and Freund Y. , Effect of Patient Gender on the Decision of Ceiling of Care: An European Study of Emergency Physicians’ Treatment Decisions in Simulated Cases, European Journal of Emergency Medicine. (2024) 31, no. 6, 423–428, 10.1097/MEJ.0000000000001176. 39350568

[26] Coisy F. , Desbrosses C. , Markarian T. et al., Thirty-Day Survival Rate of Patients Having a Treatment Withholding or Treatment Withdrawal Decision in the Emergency Department: A Retrospective Monocentric Study, Geriatrics and Gerontology International. (2025) 25, no. 4, 528–534, 10.1111/ggi.70013. 40017164

[27] Pallarès N. , Inouzhe H. , Straw S. et al., Development and Validation of a Model to Predict Ceiling of Care in COVID-19 Hospitalized Patients, BMC Palliative Care. (2024) 23, no. 1, 10.1186/s12904-024-01490-8. PMC11250965 39010044

[28] Slack P. and Rose A. , Ceiling of Care Decisions at an Older Person’s Mental Health Unit in Gloucestershire, BMJ Quality Improvement Reports. (2015) 4, no. 1, 10.1136/bmjquality.u207618.w3050. PMC4645949 26734377

[29] Straw S. , McGinlay M. , Drozd M. et al., Advanced Care Planning During the COVID-19 Pandemic: Ceiling of Care Decisions and Their Implications for Observational Data, BMC Palliative Care. (2021) 20, no. 1, 1–11, 10.1186/s12904-021-00711-8. PMC7797882 33430850

[30] Hux R. , For and Against the Four Principles of Biomedical Ethics, Clinical Ethics. (2013) 8, no. 2-3, 39–43, 10.1177/1477750913486245.

[31] Misselbrook G. P. , Jackman D. , Vora C. , Briant-Evans T. , and Wilkinson A. , Implementing and Improving the Respect Process Within Medical and Orthopaedic Departments of a District General Hospital, Progress in Palliative Care. (2020) 28, no. 4, 254–259, 10.1080/09699260.2019.1677985.

[32] Piers R. , Pautex S. , Cano L. R. et al., Goals of Care Discussions and Treatment Limitation Decisions in European Acute Geriatric Units: A One-Day Cross-Sectional Study, Age and Ageing. (2025) 54, no. 2, 1–10, 10.1093/ageing/afaf026. PMC11836419 39967416

[33] Vranas K. C. , Lin A. L. , Zive D. et al., The Association of Physician Orders for Life-Sustaining Treatment With Intensity of Treatment Among Patients Presenting to the Emergency Department, Annals of Emergency Medicine. (2020) 75, no. 2, 171–180, 10.1016/j.annemergmed.2019.05.008. PMC6928444 31248675

[34] Resuscitation Council UK , Respect for Healthcare Professionals, 2025, https://www.resus.org.uk/respect/respect-healthcare-professionals.

[35] Fritz Z. , Slowther A.-M. , and Perkins G. D. , Resuscitation Policy Should Focus on the Patient, Not the Decision, British Medical Journal. (2017) 356, 10.1136/bmj.j813. PMC5330195 28246084

[36] Bruckel J. , Mehta A. , Bradley S. M. et al., Variation in do-not-Resuscitate Orders and Implications for Heart Failure Risk-Adjusted Hospital Mortality Metrics, Journal of the American College of Cardiology: Heart Failure. (2017) 5, no. 10, 743–752, 10.1016/j.jchf.2017.07.010. PMC7552359 28958349

[37] Walkey A. J. , Weinberg J. , Wiener R. S. , Cooke C. R. , and Lindenauer P. K. , Hospital Variation in Utilization of Life-Sustaining Treatments Among Patients With Do Not Resuscitate Orders, Health Services Research. (2018) 53, no. 3, 1644–1661, 10.1111/1475-6773.12651. PMC5980340 28097649

[38] Perkins G. D. , Perkins G. , Griffiths F. et al., Do-Not-Attempt-Cardiopulmonary-Resuscitation Decisions: An Evidence Synthesis, Health and Social Care Delivery Research. (2016) 4, no. 11, 1–154, 10.3310/hsdr04110.

[39] Pitcher D. , Fritz Z. , Wang M. , and Spiller J. A. , Emergency Care and Resuscitation Plans, BMJ. (2017) 356, 10.1136/bmj.j876. 28246080

[40] Yuen J. K. , Reid M. C. , and Fetters M. D. , Hospital do-not-Resuscitate Orders: Why They Have Failed and How to Fix Them, Journal of General Internal Medicine. (2011) 26, no. 7, 791–797, 10.1007/s11606-011-1632-x. PMC3138592 21286839

[41] Clemente J. R. , Hess P. , Oca C. et al., The Effect of a “Do Not Resuscitate”(DNR) Status on Patient Care: A Descriptive Survey on the Perceptions of ICU and Medical/Surgical Nurses, International Journal of Critical Care. (2023) 17, no. 2, 39–53, 10.29173/ijcc62.

[42] Moffat S. , Skinner J. , and Fritz Z. , Does Resuscitation Status Affect Decision Making in a Deteriorating Patient? Results From a Randomised Vignette Study, Journal of Evaluation in Clinical Practice. (2016) 22, no. 6, 921–927, 10.1111/jep.12559. PMC5111586 27237130

[43] Mehlis K. , Bierwirth E. , Laryionava K. , Mumm F. , Heussner P. , and Winkler E. C. , Late Decisions About Treatment Limitation in Patients With Cancer: Empirical Analysis of End-of-Life Practices in a Haematology and Oncology Unit at a German University Hospital, ESMO Open. (2020) 5, no. 5, 10.1136/esmoopen-2020-000950. PMC7592262 33109628

[44] Van Erp I. A. , Van Essen T. , Kompanje E. J. et al., Treatment-Limiting Decisions in Patients With Severe Traumatic Brain Injury in the Netherlands, Brain and Spine. (2024) 4, 10.1016/j.bas.2024.102746. PMC10951765 38510637

[45] Borsellino P. , Limitation of the Therapeutic Effort: Ethical and Legal Justification for Withholding and/or Withdrawing Life Sustaining Treatments, Multidisciplinary Respiratory Medicine. (2015) 10, no. 1, 10.1186/s40248-015-0001-8. PMC4335772 25705381

[46] Mercadante S. , The Paradigm Shift from End of Life to Preemptive Palliative Care in Patients With Cancer, Cancers. (2022) 14, no. 15, 10.3390/cancers14153752. PMC9367388 35954417

[47] Cruz-Carreras M. , Chaftari P. , and Viets-Upchurch J. , Advance Care Planning: Challenges at the Emergency Department of a Cancer Care Center, Supportive Care in Cancer. (2018) 26, no. 2, 585–588, 10.1007/s00520-017-3870-x. PMC5752753 28918550

[48] Brom L. , De Snoo-Trimp J. C. , Onwuteaka-Philipsen B. D. , Widdershoven G. A. M. , Stiggelbout A. M. , and Pasman H. R. W. , Challenges in Shared Decision Making in Advanced Cancer Care: A Qualitative Longitudinal Observational and Interview Study, Health Expectations: An International Journal of Public Participation in Health Care and Health Policy. (2017) 20, no. 1, 69–84, 10.1111/hex.12434. PMC5217936 26669902

[49] Broadhurst H. L. , Droney J. , Callender T. , Shaw A. , and Riley J. , Advance Care Planning: The Impact of Ceiling of Treatment Plans in Patients With Coordinate My Care, BMJ Supportive & Palliative Care. (2019) 9, no. 3, 267–270, 10.1136/bmjspcare-2017-001414. 29572344

[50] Shapiro J. , Acholonu C. , and Madrigal V. , Ethics in the Emergency Department: Withholding or Terminating Resuscitation, Pediatric Emergency Care. (2025) 41, no. 8, 661–666, 10.1097/PEC.0000000000003423. 40742225

[51] Beckwith S. , Dickinson A. , and Kendall S. , The “Con” of Concept Analysis: A Discussion Paper Which Explores and Critiques the Ontological Focus, Reliability and Antecedents of Concept Analysis Frameworks, International Journal of Nursing Studies. (2008) 45, no. 12, 1831–1841, 10.1016/j.ijnurstu.2008.06.011. 18715562

[52] Byambasuren O. , Myooran J. , Virk A. et al., Advance Care Planning in Patients With Suspected or Proven COVID: Are we Meeting our Own Sandards?, American Journal of Hospice and Palliative Medicine. (2024) 41, no. 11, 1358–1362, 10.1177/10499091231218476. 38032286

[53] De Decker L. , Beauchet O. , Gouraud-Tanguy A. , Berrut G. , Annweiler C. , and Conte P. , Treatment-Limiting Decisions, Comorbidities, and Mortality in the Emergency Departments: A Cross-Sectional Elderly Population-Based Study, The Journal of Nutrition, Health & Aging. (2012) 16, no. 10, 914–918, 10.1007/s12603-012-0414-4. PMC12878261 23208032

[54] Haimovich A. D. , Numata K. , Wolozin J. et al., Advance Care Planning Engagement of Older Adults in the Emergency Department, American Journal of Hospice and Palliative Medicine. (2025) 1, 606–612, 10.1177/10499091251338252. PMC12377290 40271984

[55] Holmes D. , Taylor R. , and Carberry M. , Using Treatment Escalation and Limitation Plans to Ensure Appropriate Emergency Care, Nursing Times. (2019) 115, no. 12, 38–41, https://research.ebsco.com/linkprocessor/plink?id=ea5a9fce-3476-3ec1-9f96-49a2ebcbd9a2.

[56] Knight T. , Malyon A. , Fritz Z. et al., Advance Care Planning in Patients Referred to Hospital for Acute Medical Care: Results of a National Day of Care Survey, EClinicalMedicine. (2020) 19, 10.1016/j.eclinm.2019.12.005. PMC7005412 32055788

[57] Lim C. E. D. , Ng R. W. C. , Cheng N. C. L. , Cigolini M. , Kwok C. , and Brennan F. , Advance Care Planning for Haemodialysis Patients, Cochrane Database of Systematic Reviews. (2016) 2016, no. 7, 10.1002/14651858.CD010737.pub2. PMC6458029 27457661

[58] Walzl N. , Sammy I. A. , Taylor P. M. , Smith J. E. , and Lowe D. J. , Systematic Review of Factors Influencing Decisions to Limit Treatment in the Emergency Department, Emergency Medicine Journal. (2022) 39, no. 2, 147–156, 10.1136/emermed-2019-209398. 33658272

