# Supplementary material for: Ceiling of Care in Acute Care Settings: A Concept Analysis
Source: J Nurs Manag. 2026 Sep 22;2026:6624553. doi: 10.1155/jonm/6624553 (PMC13598369; doi:10.1155/jonm/6624553)
Supplement: Supplementary file 2 [file JONM-2026-6624553-s002.doc]

**Table S1 Definition of Ceiling of Care and Related Terms**

| **Term** | **Author** | **Definition** |
| --- | --- | --- |
| **Ceiling of care** | (Pallares et al., 2023) | "Therapeutic ceiling was defined as the maximum therapeutic effort to be offered to a subject on the basis of their age, their associated comorbidities, and the expected clinical benefit in relation to the availability of available resources." |
|  | (Pallarès et al., 2024) | "Therapeutic ceiling of care is the maximum level of care deemed appropriate to offer to a patient based on their clinical profile and therefore their potential to derive benefit, within the context of the availability of resources. |
|  | (Slack & Rose, 2015) | Not explicitly defined in the article, but operationalised through four-level system adapted from acute hospital policy:  Attempt CPR, for full active treatment including transfer to general hospital  DNACPR but for full active ward-based care including transfer to general hospital  DNACPR, care to be given at OPMHS unit only  DNACPR, do not transfer to general hospital and consider palliative referral |
|  | (Straw et al., 2021) | Ceiling of Care levels: "Level one care was hospitalisation without need for organ support (but including oxygen therapy) and delivered in a ward setting; level two care was single organ support (usually CPAP), but excluding mechanical ventilation, and delivered in either in a ward, high-dependency unit or ICU setting; level three care was multi-organ support or mechanical ventilation and delivered on the ICU" |
|  | (Vromant et al., 2024) | The concept of a Ceiling of Care in emergency medicine involves setting a maximum level of care considered appropriate for a patient, guided by healthcare teams in collaboration with the patient's and family's beliefs and values" |
|  | (Walzl et al., 2019) | Ceiling of treatment is considered to be the predetermined highest level of intervention deemed appropriate by a medical team, aligning with patient and family wishes, values and beliefs. |
| **Advance Care Planning (ACP)** | (Byambasuren et al., 2024) | ACP is a process that uses a person's values, beliefs and preferences to guide medical decision-making |
|  | (Cruz-Carreras et al., 2018) | "Advance care planning (ACP) refers to a process that permits patients and their family members to discuss with healthcare providers the patient's wishes for end-of-life care" |
|  | (Haimovich et al., 2025) | "Advance care planning (ACP) is the process of making decisions about medical treatment and substitute decision-making towards the end of life" |
|  | (Knight et al., 2020) | "A process 'enabling individuals to define goals and preferences for future medical treatment and care, to discuss these goals and preferences with family and healthcare providers, and to record and review these preferences if appropriate'" |
|  | (Lim et al., 2016) | "Advance care planning involves planning for future healthcare decisions and preferences of the patient in advance while comprehension is intact." |
| **Treatment-Limiting Decision** | (De Decker et al., 2012) | "The treatment-limiting decision was defined as a predetermined choice to not implement therapies that would otherwise be deemed necessary (i.e., endotracheal intubation, mechanical ventilation, intravenous fluid replacement, massive transfusion [>3 units of packed red blood cells], vasopressor infusion, cardiopulmonary resuscitation, and renal replacement therapy)" |
|  | (Piers et al., 2025) | "A treatment limitation decision was defined as a written order specifying the intensity of treatment and grouped into four subcategories: (i) no Cardio-Pulmonary Resuscitation (CPR) only; (ii) no CPR and no intubation [other Intensive Care Unit (ICU) treatment possible]; (iii) no ICU treatment, only ward based medical treatment; (iv) no life prolongation and focus on comfort care. |
|  | (Walzl et al., 2022) | NA |
| **Physician Orders for Life-Sustaining Treatment (POLST),** | (Vranas et al., 2020) | The Physician Orders for Life-Sustaining Treatment (POLST) program was developed in the 1990s in Oregon to help prevent the provision of unwanted medical interventions to patients with advanced illness or frailty who are approaching the end of life" |
| **Treatment Escalation or Limitation Plan (TELP)** | (Holmes et al., 2019) | "A treatment escalation or limitation plan (TELP)...is a communication tool that determines whether interventions are likely to be appropriate or inappropriate (potentially futile, harmful and/or contrary to patient's wishes in the event of clinical deterioration)" |
| **Recommended Summary Plan for Emergency Care and Treatment ReSPECT** | (Misselbrook et al., 2020) | "The ReSPECT is a process that creates personalized recommendations for clinical care in a future emergency in which they are unable to make or express choices. It provides healthcare professionals with a summary of recommendations to help them to make immediate decisions about that person's care and treatment." |

Note: CPR, Cardiopulmonary Resuscitation; DNACPR, Do Not Attempt Cardiopulmonary Resuscitation; OPMHS, Older People's Mental Health Service
